# From leader to peer – specializing physicians’ understanding of their multiple positions in interprofessional health care teams

**DOI:** 10.1080/10872981.2025.2534058

**Published:** 2025-07-18

**Authors:** Emma Sallinen, Leena Mikkola, Stephanie Fox, Heli Parviainen

**Affiliations:** a Faculty of Information Technology and Communication Sciences, Tampere University, Tampere, Finland; b Department of Communication, University of Montreal, Montreal, Canada; c Faculty of Social Sciences, Tampere University, Tampere, Finland

**Keywords:** Interprofessional communication, residents, leadership, professional identity formation, positioning theory

## Abstract

**Introduction:**

This exploratory qualitative study aims to uncover the identity work in interprofessional (IP) team interaction to understand specializing physicians’ (SP) professional identities by analyzing how they position themselves in relation to others through the lens of positioning theory.

**Method:**

The data for this study consist of 65 self-reflexive essays written by SPs during their mandatory leadership studies.

**Results:**

Altogether, five distinct physician positions (peer, coordinator, leader, medical expert, and decision-maker) and two distinct storylines (teamwork as communication vs. teamwork as an organizational tool) were identified during the positioning analysis.

**Discussion:**

The wide range of physician positions reveals how diversely and dynamically SPs adapt leadership as part of their professional identity and reframes future studies to explore *how* SPs construct the dimensions of their professional identity rather than whether they do so.

## Introduction

Specializing physicians (SPs) are often expected to assume leadership within interprofessional (IP) teams [1]. This issue of leadership within IP teams is critical, as IP teamwork has been demonstrated to be an effective way to organize expertise within health care (HC) teams [2], benefiting both patients and HC personnel [3]. However, Roten et al. [4] have highlighted that the demands of IP work present identity challenges for SPs, who may struggle to understand how to fulfill their expected managerial leadership roles in IP teams [4]. While SPs’ professional identity formation (PIF) begins during their basic education [5], it is during their specialization training that a deeper understanding of the interdependence among HC professionals starts to take shape. It is also at this stage that SPs begin to learn about the leadership and administrative roles required by their profession [4]. This presents an identity challenge, which Berghout et al. [6] have referred to as the dual-identity problem as SPs may find it difficult to integrate new leadership identity into their professional identity (e.g [7–9]. To promote high-quality care through effective IP teamwork, it is essential to understand how SPs perceive their position within IP teams, particularly in relation to their professional and leadership identities.

## Physician’s PIF and IP teams

PIF has been widely adopted as a theoretical framework to study the identity development of medical professionals during their education and careers (see e.g [10]. PIF is often viewed as a straightforward process, where individuals enter medical school with pre-existing identities and the desire to join the physician community and pursue a specific medical practice [11]. Through this process, their *professional values, actions, and aspirations* gradually evolve [12]. Although PIF has been conceptualized as progressing through different stages, often represented in a pyramid (knowledge, competence, performance, and action, from bottom to top [13, 14], this study focuses instead on the *interactional nature* of identity development. We emphasize that professional identity is a constantly negotiated construct. Thus, our approach to professional identity is socio-constructionist (e.g [7]. We argue that professional identity is not something physicians graduate with but something that develops over time and through interactions with others in HC organizations [13,15]. Therefore, we aim to broaden studies of physicians’ PIF to include an understanding of how PIF occurs within IP teams.

Physician identity is deeply rooted in the patient – physician relationship [6]. As a result, physicians often feel the need to protect their professional identity from challenges that might threaten their professional autonomy, such as increasing managerial and leadership requirements [16]. According to Walraven et al. [17], SPs feel that their education does not equip them with the necessary competencies to participate in, let alone lead, IP team meetings. This suggests that the transition into specialization and becoming an expert in their chosen field represents a stressful and even burdensome identity shift for SPs [4]. However, regardless of whether SPs feel prepared to lead, they are expected to do so within IP teams, even early in their careers [4]. To explore how SPs’ professional identity is in flux within IP teams, we now turn to the positioning theory.

## Theoretical background: positioning theory

Positioning theory (PT) is a socio-constructionist framework proposing that communication and positioning have a dynamic, identity-shaping nature [18]. It explains how individuals deliberately and mutually position themselves in relation to others during interactions as a means of expressing their identity [19,20]. A key premise of PT is that identities are made socially and subjectively meaningful by distinguishing between sameness and otherness (ibid [21,22]. Position, in this context, refers to the relational stance one takes in relation to others, describing how rights and responsibilities – specifically interactional rights and responsibilities – are distributed in these relations, enabling meaningful actions in social interactions [23].

The foundation of PT lies in a triangular framework of positioning: position, speech act, and storyline [18]. *Positions* define the boundaries (rights and responsibilities) of socially acceptable actions within interactions [20]. For example, an SP may occupy multiple positions within IP teams, such as a colleague or a novice, each conferring distinct interactional rights and responsibilities. In PT, *speech acts* [19,24] refer to the actual communicative actions deemed significant by participants, which help make their own and others’ actions socially determinable. This highlights the social nature of speech acts, which are contextually grounded and informed by moments of interaction, such as the elaborative questions a physician asks of nurses in an IP team. *Storylines* represent the contextual and situational factors that influence the appropriateness and availability of different positions [25]. For instance, organizational norms shape how physicians communicate in different situations, such as during weekly team meetings.

This study aims to understand SPs’ professional identity work through the lens of PT by analyzing how they position themselves in relation to others within the frame of IP team interactions. We will examine how SPs perceive the distribution of rights and responsibilities in their relationships with the team, i.e., other professionals, and how these interactional rights and responsibilities position everyone involved and influence their participation in patient care. Thus, we consider the positions as identity positions (see [19], focusing on the meanings SPs construct regarding their relationship with the IP team. We do not seek to explore the underlying factors (i.e., gender or specialization) that might explain why certain positions emerge in specific situations, but rather, we aim to explore the full range of positions that can be identified from SPs’ experiences. Therefore, we pose the following research question:

RQ: What kinds of leadership positions do SPs construct in IP teams as a part of their professional identity?

## Method

### Research design

The overall research design of this study is qualitative, employing a theory-driven approach. It aims to examine the descriptions of positioning and generate insights to enhance understanding of IP team communication, thereby contributing to leadership studies in medicine education. The study seeks to broadly describe the range of possible identity positions and is thus descriptive in nature. Consequently, the analysis does not focus on areas of specialty or demographic features. The data consisted of 65 individual essays by SPs. The data were collected as part of a communication module within a specialist training program at a Finnish university offering a specialist curriculum. Thus, the study was conducted within a university setting and the data collection happened as a part of specializing studies in medical school. The participants reflect their experiences in real life situations in different health care organizations, but the study does not include any information about the health care facilities or observatory data.

The theoretical framework of data analysis was PT [18], with speech acts, positions, and storylines identified in the texts. PT has primarily been applied to naturally occurring data to explore how positions are situationally and contextually negotiated in interaction [18]. However, it has also been successfully applied to the study of monologic written texts, such as diary entries (e.g [26, 27]., and interviews [28]. As the data in this study consist of self-reflexive essays, this research represents a novel application of PT to this type of data.

### Participants

In Finland, specialist training in medicine constitutes a postgraduate degree offered at any of the five medical faculties. Completion of the degree typically requires approximately 5 to 6 years of study (300–360 credits according to European Credits Transform System, or ECTS) and includes medical practice, nine months of service in primary HC centers, theoretical coursework, management studies, and successful passing of a national written examination (for further details, see [29,30]. Each specialization program includes 10 ECTS of compulsory management studies covering organizational management and leadership, the social and HC system, human resources management, leadership interaction and organizational communication, HC economics, legislation, and data management. Additionally, trainees may take up to 20 ECTS of elective management studies to supplement the required coursework.

While undertaking specialization studies, participants work in public HC clinics across Finland once a month to complete their mandatory leadership studies. The communication module emphasizes leadership and workplace communication, as well as media-related topics. It includes pre-reading materials, lectures, and an essay assignment. SPs are free to choose the timing of their mandatory leadership courses, which are common across all specializations. As a result, participants vary in experience and areas of specialty. The study participants worked in both hospital organizations and health care centers with many having experience in both settings. As our aim was to explore an overview of the possible positions SPs have faced, we did not gather detailed personal information. Nevertheless, the data reflect a wide range of specialty areas (a total of 16 different specialties were mentioned in the essays), from psychiatry to anesthesiology and from surgery to neurology and pediatrics. However, given the limited number of SPs from each specialty, meaningful comparisons across specialties are not possible. All participants reported working in various types and configurations of IP teams, highlighting the significance of teamwork, collaboration, and communication in their professional roles.

### Data collection

The data consisted of learning assignments given during a lecture by the fourth author, who was coordinating the communication module. Participants were instructed on how to complete the essay and how to provide their consent should they choose to participate in the study. To complete their essays, participants were asked to describe their views on IP collaboration and leadership in IP settings, reflecting on their own experiences. The essay instructions were open-ended, giving SPs the freedom to explore their experiences of IP communication in the workplace. The concept of the IP team was introduced in broad terms, allowing participants to write from the perspective of their own current and previous workplaces. Consequently, the data reflected a variety of team types and collaboration with health and social care professionals, such as nurses, nurse practitioners, physiotherapists, psychologists, ward clerks, and specialists from other fields of medicine.

While no strict word limit was set, they were asked to write a minimum of three pages. SPs had two weeks to complete their essays after the lectures of the module had concluded, submitting them as a home assignment. The essays ranged from 2–5 A4 pages, single-sided, with 1.5 line spacing and a font size of 12.

Writing the essay was a mandatory component of the module, but participation in the study was entirely voluntary. Choosing not to participate had no impact on the completion of the module, workplace learning, or specialization training program. All participants in the module were invited to take part in the study, regardless of their various backgrounds or levels of experience, and no exclusion criteria were applied. The essays were submitted via the online learning platform Moodle. Although the instructions guided participants to focus on IP collaboration, most SPs chose to write about IP teamwork. This highlights the ambiguity of the term ‘collaboration’ and how ‘collaboration’ and ‘teamwork’ are often treated as synonyms. Since the SPs predominantly used the term ‘teamwork’ over ‘collaboration,’ the analysis focused on teamwork as defined by the participants.

### Data analysis

The analysis in this study was inspired by thematic analysis but was based on the triangular framework of PT outlined in the previous section: position, speech act, and storyline [18]. By analyzing these three aspects of interaction, it is possible to gain insights into how individuals position themselves within interaction. The analysis was conducted using the Atlas.ti software.

The first author carried out the analysis, but interpretations and coding decisions were discussed collaboratively with the co-authors. Initially, the data were read multiple times to establish a comprehensive understanding. Subsequently, all content related to interprofessionality, teamwork, leadership, and the role of the physician was extracted to create a focused dataset. Following the PT framework, the first phase of analysis involved identifying all the speech acts in the data. Given that the data consisted of self-reflexive essays, speech acts were interpreted as meaning units (see [31]. In this context, a meaning unit was defined as a coherent segment of text where the physicians positioned themselves in relation to the IP team, for example, ‘I believe that a physician’s responsibilities include treating both colleagues and patients with respect.’

Next, the meaning units were coded based on *how* SPs positioned themselves in relation to the IP team. Positions were identified through the use of personal pronouns [32], such as ‘ … as a physician, in these situations, ***I*** trust that the nurses take care of ***my*** safety, too … ,’ and by examining *how* SPs described their relational stance with regard to *others* [33], for instance, ‘ … I’m not *ordering anyone to do anything*; instead, *we discuss things together* … ’ The *others* in these statements referred to the rest of the IP team and members of HC organizations. As SPs described their own roles, they simultaneously positioned the other team members [20]. In total, over 600 speech acts were identified. The initial round of open coding led to the identification of eight different identity positions: peer, coordinator, chairperson, leader, professional, medical expert, unsure, and decision-maker. However, some codes overlapped, and the positions were not entirely distinct. Therefore, the coding was reviewed and refined in collaboration with all the authors to ensure systematic consistency. As a result, the codes were consolidated into five exclusive identity position categories, each representing how SPs viewed themselves in relation to others on the IP team. It is important to note that multiple position categories were typically present in a single essay, illustrating the situational and contextual nature of positioning [18].

After finalizing the categories, the first author reviewed the original data to validate and confirm the findings. The analysis ultimately yielded five identity position categories. Following this, storylines were identified by further analyzing how the position categories related to one another. Two dominant storylines emerged from the data: one conceptualized IP teamwork as communication among team members, while the other framed IP teamwork as a functional organizational tool. Both position categories and storylines will be described in greater detail in the Results section.

### Ethical considerations

The ethical principles of scientific study were strictly followed according to the Finnish National Board of Integrity throughout the study (Finnish Code of Conduct for Research Integrity [34]. Informed consent was obtained by providing participants with information about the study before requesting their participation. Written consent was acquired for the use of their essays in the research. All personal data, such as names and contact information, was removed prior to data analysis to ensure the privacy of the participants. While some participants mentioned their specialties, this information was included in the datasheet, as it was not possible to identify them solely based on this information.

## Results

Five distinct identity positions were identified in the SPs’ essays: 1) peer, 2) coordinator, 3) team leader, 4) medical expert, and 5) decision-maker. These positions represent the SPs’ understanding of their own roles within the IP team, or how they perceive themselves in relation to their colleagues. Additionally, as mentioned, two overarching storylines emerged from the data: 1) teamwork as communication and 2) teamwork as an organizational tool. SPs were observed moving dynamically between these positions. The novelty of our results lies in conceptualizing physician leadership as constituted through team interaction, and in applying positioning theory as a methodological lens to reveal the interactional and dynamic nature of leadership positions – an approach that, to our knowledge, has not been previously employed in this context. The interactional rights and responsibilities that define the boundaries of identity positions, along with the storylines they produce, are described in Table 1, from both SPs’ perspectives and their perceptions of the others’ viewpoints.

**Table 1.** Distribution of rights and responsibilities in SPs’ leadership positions and the storylines constructed by these positions in Finland in 2018.

Position/storyline	Specializing physicians’ rights	Others’ rights	Specializing physicians’ responsibilities	Others’ responsibilities
**Peer** The storyline of teamwork as communication is dominant	…to participate in processes that impact patient care.	…to participate in processes that impact patient care.	…to ensure the shared IP team goal of quality patient care. …to report one’s actions to the IP team.	…to ensure quality patient care. …to report one’s actions to the IP team.
**Coordinator** The storyline of teamwork as communication is emphasized	…to participate in and coordinate the processes that impact patient care. …to make informed decisions regarding patient care.	…to participate in processes that impact patient care.	…to ensure the shared IP team goal of quality patient care with effective team processes and outcomes. …to coordinate the team’s shared expertise. …to report one’s actions to the IP team.	…to ensure quality patient care. …to report one’s actions to the IP team.
**Team leader** The storyline of teamwork as an organizational tool gets introduced	…to lead processes that impact patient care. …to make informed decisions regarding patient care.	…to assist in processes that impact patient care.	…to ensure the team’s performance regarding the quality of patient care and teamwork. …to answer to their own supervisor.	…to assist the physician with processes that impact patient care. …to report one’s actions to the physician.
**Medical expert** The storyline of teamwork as an organizational tool gets emphasized	…to utilize the IP team in processes that impact patient care and the right to decide how these processes are executed. …to consult other members of the IP team and determine whose expertise is important. …to make decisions regarding patient care.	…to assist in processes that impact patient care if one has the knowledge and skills.	…to ensure the team’s and its members’ performance and outcomes. …to avoid errors in processes that impact patient care.	…to assist the physician with processes that impact patient care. …to report one’s actions to the physician.
**Decision maker** The storyline of teamwork as an organizational tool is dominant	…to utilize or dismiss the IP team in processes that impact patient care. …to consult other members of the IP team and to gather information from them. …to supervise and give instructions or orders to others on the IP team. …to make decisions over others regarding patient care.	…to assist in processes that impact patient care processes if one has the knowledge and skills.	…to be accountable for the performance and outcomes of patient care. …to answer to their own supervisor. …to ensure effective processes that impact patient care.	…to assist the physician with processes that impact patient care. …to gather information for the physician. …to report one’s actions to the physician.

### The identity position of peer

From the peer position, minimal differences exist between the rights and responsibilities of SPs and other team members. Shared responsibility and equality in patient care are emphasized, with every professional considered an essential and valuable part of the care process. All team members have an equal right to participate in processes that impact patient care, such as discussions, decision-making, and even diagnostics, by contributing their professional insights. When adopting the peer position, SPs actively construct a low-hierarchy or even non-hierarchical team, where every team member has the right to share their perspective on the situation, and everyone’s opinion is taken into consideration. The storyline of teamwork as communication is particularly strong in this position, and SPs enact it in ways that prioritize communication as the central element of teamwork. A shared goal of addressing the patient’s needs justifies the equal distribution of responsibilities and rights among team members, as demonstrated in Extract 1:

**Extract 1**. The physician’s role in interprofessional communication is, in my opinion, the same as every other profession that participates in communication: to work for the patient’s wellbeing on their behalf.

(SPO16)

As SPs acknowledge the organizational and hierarchical status that accompanies their medical responsibility, they strive to construct a more equal position by shifting from hierarchical, physician-centered thinking to a model of collaborative and joint responsibility for the patient. They do so by actively inviting others to participate, fostering an atmosphere conducive to joint decision-making, where every team member plays a role. While the tasks of each professional may differ, equality emerges through the shared commitment to patient care. This demonstrates that, although SPs recognize their organizational power and the hierarchy among professions, they consciously work to reduce these hierarchical dynamics through communication, that is, building teamwork around interaction rather than structure. By adopting the identity position of a peer, SPs deconstruct the traditional physician-led model, as described in Extract 2:

**Extract 2**. An important shift happens from the point of interprofessional leadership. Instead of traditional hierarchy, a coordinated and equal team-based collaboration emerges, like, “We are jointly responsible for our patients, and we lead the care processes together.”

(SPO04)

### The identity position of coordinator

In this identity position, the SP assumes responsibility for coordinating the IP team’s discussions and decision-making processes, with the aim of enhancing team effectiveness and outcomes. Simultaneously, SPs have the right to request consultations and seek the opinions of others by leading team conversations and guiding the processing of information. While the responsibility for delivering quality care is shared, SPs are tasked with coordinating, chairing, and, where necessary, making decisions. The coordinator’s role differs from that of a peer. As coordinators, SPs are responsible for ensuring that the team functions efficiently, acting as chairpersons or designated coordinators. In this sense, the SP is not a supervisor but a manager who facilitates the team’s operations. Nevertheless, decision-making remains a shared process, as shown in Extract 3:

**Extract 3**. This does not mean that the SP is the supervisor of these professions. Mainly, I think that the role of a physician is more about keeping all the strings in one’s hands and piloting with open discussions. Because leadership matters to the other professions, they can plan their own work better.

(SPO16)

SPs recognize the expertise of other professionals and acknowledge that their own responsibility lies in knowing who possesses the most relevant knowledge in a given situation. Therefore, they have the right to decide whose expertise should be prioritized, which may, in some cases, lead to decisions being made without consulting every team member. Simultaneously, SPs have the right to access all the information related to the case from the team. The storyline of teamwork as communication remains dominant in the coordinator position, as SPs emphasize collaborative dialogue over their organizational authority. While responsibility for patient care is shared among team members, SPs serve as coordinators within the care process. In this role, interdependence between the SP and the IP team remains high, as demonstrated in Extract 4:

**Extract 4**. On the other hand, you must be able to listen, negotiate, and respect opinions you don’t necessarily agree with. Many times, physicians are the ones who end up with the responsibility of being the so-called chairperson, to manage the whole situation, to make summaries, to organize, and to make follow-up plans.

(SPO29)

### The identity position of team leader

When occupying the identity position of team leader, SPs’ rights and responsibilities are bound to their organizational status: They are responsible for the overall performance of the IP team and for overseeing their subordinates. SPs hold the right and duty to monitor and manage the processes of the IP team. However, they are also accountable to their own supervisors, which places them within the organizational hierarchy. Indeed, during their specialization training, there is always someone above them with greater authority to intervene in the IP team’s processes. In some cases, SPs position themselves outside the IP team by framing themselves as parts of an HC organization and a leader – subordinate chain that defines their rights and responsibilities. This contrasts with the coordinator position: as coordinators, they operate within the team; as team leaders, they are embedded within the broader organization, reporting their actions to a superior.

Even though the SP is responsible for managing the team, responsibility for patient care remains shared. All team members have both the right and the obligation to contribute to care by applying their respective knowledge and expertise to the team’s decision-making. The identity position of the team leader is summarized in Extract 5:

**Extract 5**. The physician is often thought of as the “leader” in interprofessional communities. Sure, the physician makes a medical assessment of the situation and takes most time the biggest responsibility for the big picture. Anyway, you cannot think that the physician always dictates to other professions what they should do.

(SPO42)

SPs acknowledge the value of communication within the IP team, but they are also expected by the HC organization to utilize the team as a tool. As a result, the storyline of teamwork as communication is interrupted by that of teamwork as an organizational instrument. Although SPs have the right to manage the diagnostic process, they do not have the right to question, micromanage, or dictate how others should perform their respective roles.

### The identity position of medical expert

This identity position emphasizes the SPs’ medical responsibility for the patient. From this standpoint, an SP identifies more strongly with their specialty and profession – and with their supervising physician – than with the team. This underscores that the SP is ultimately responsible for the patient’s care, with legal accountability emphasized. When adopting the medical expert position, SPs have the right and are encouraged to draw upon the IP team’s insights to aid in diagnosis. Nonetheless, the ultimate responsibility for the patient and the care process lies with the SP. This grants them the authority to make decisions about the use of IP teamwork and its execution. They are also entitled to determine which information and contributions from the team are important and should be considered in decision-making. Thus, the storyline of teamwork as an organizational tool becomes dominant. In this frame, the team is a tool the SP may choose to use if they deem it necessary. Whereas in the storyline of IP teamwork as communication, the physician is seen as a member of the team, in this position, the physician is rather an outsider and not active participant of the teamwork dynamic.

Other team members have the right and responsibility to participate only when they have something specific to contribute or when the SP consults them. With this ultimate responsibility, SPs are partly accountable for the actions of everyone on the team. If another team member makes an error in patient care, the SP is the one who assumes full responsibility. As a result, SPs often find themselves double-checking others’ work since they are held accountable for the team’s overall performance. To avoid errors, the team depends on the SP’s effort and oversight. Extract 6 captures how SPs describe their position ‘at the top of the food chain,’ suggesting that their power position can also lead to professional isolation:

**Extract 6**. At the top of the food chain, physicians’ position is quite lonely when making the final decisions and taking responsibility for everything, even the accomplishments of supportive groups.

(SPO10)

This position is primarily assigned by the organization rather than emerging from team communication with a non-negotiable leadership role within the IP team. As this position is imposed by the organization, with expectations for the SP to act as a medical professional, the SP’s ability to influence their own role is very limited. While SPs may be reluctant to accept this position, their organizational status prevents them from openly challenging it. Instead, they are compelled to assume and perform this identity in front of the IP team – a task they often find difficult, as illustrated in Extract 7:

**Extract 7**. Responsibilities weigh young physicians down when multiple things must be taken care of very independently. At the end of the day, responsibility for everything, including the big picture, lies upon them. This role is not easy; in fact, it’s even ruthless.

(SPO10)

SPs struggle with the demands of leading IP teamwork as a part of patient care, particularly since they are still early in their career, both as physicians and leaders. At times, they even feel helpless when they are expected to lead IP collaboration without adequate preparation or practical leadership experience, as shown in Extract 8:

**Extract 8**. The physician might end up as a leader straight out of medical school, even as the youngest and most inexperienced of the team and one who holds no interest or competence in leadership.

(SPO60)

### The identity position of decision-maker

From this identity position, SPs are viewed as the sole and autonomous decision-makers within the IP team. By accepting this position, they also accept ultimate responsibility for patient care. As such, they have full authority to utilize or disregard the IP team, which exists to serve the SP’s needs when consultation is required. Here, the storyline of teamwork as a tool becomes dominant, and, aligning closely with physician-centered practice, teamwork is not something constituted by the team but something the physician deploys. Other team members do not participate in decision-making. Their role is limited to collecting information so the SP can perform their tasks effectively. When SPs adopt this identity, they also assume a highly hierarchical stance aligned with organizational structures. SPs are not obligated to explain or justify their decisions to other team members – and only in some rare instances, to their supervisor. Decision-making becomes authoritarian, based solely on the SP’s clinical judgment, as described in Extract 9:

**Extract 9**. The physician gathers prerequisites from the patient or next of kin, makes the clinical status, assesses the big picture, consults colleagues about the situation, and gives the patient the follow-up treatment plan.

(SPO09)

This position grants the right to give instructions to others – a right coupled with the responsibility to lead and improve the team’s efficiency. This hierarchical authority differentiates the identity of a decision-maker from that of a team leader and a medical expert. In the medical expert position, SPs tend to resist or question their imposed leadership role; however, as decision-makers, they have come to accept this position in relation to the HC organization.

SPs are responsible for ensuring that everyone makes the correct decisions during information gathering and patient care processes and for monitoring these actions. Meanwhile, the role of others is to support the SP by handling simpler and less critical tasks, thereby allowing the SP to focus on diagnosis. Extract 10 shows how SPs, while recognizing the complementary nature of roles in IP teams, nonetheless reproduce traditional hierarchies when assuming the identity position of decision-maker:

**Extract 10**. Even though the job description and areas of expertise of physicians and nurses are partly clearly different and partly complementary, the physicians’ job entails giving direct instructions to nurses.

(SPO06)

## Discussion

This study aimed to examine identity work within IP team interaction and to understand SPs’ leadership identities by analyzing how they position themselves in relation to others. Five distinct physician positions were identified: peer, coordinator, team leader, medical expert, and decision-maker. These positions were defined according to PT [19]. Multiple positions were identified in each essay, indicating that SPs’ positions are highly situational. This suggests that they must navigate between these sometimes-conflicting roles in their work. In Finland, specialist training includes structured leadership education integrated into clinical practice, offering shared learning across specialties and emphasizing the situational nature of physician leadership. The identified positions represented two overarching storylines: teamwork as communication and teamwork as an organizational tool. As positions ranged from a fully team-centered approach with shared *interactional* rights and responsibilities to a highly physician-centered model characterized by authoritarian distribution of rights and responsibilities, the storylines can be recognized behind them. It is crucial to notice that these positions do not refer to formal *professional* rights and responsibilities. Identity positions do not override the legal duties physicians hold in patient care, nor do they negate the distinct skills and roles of other IP team members. Instead, these interactional rights and responsibilities reflect how IP teams negotiate decisions about patient care based on the identity positions constructed and enacted in each situation.

### Five leadership categories

The range of identity positions demonstrates how diversely and dynamically SPs construct their professional identity and find their own ways to act as the leaders they are expected to become during their specialization training.

However, as these positions differ fundamentally, they may sometimes contradict or even struggle with one another. We claim that the most significant clashes occur when SPs are expected to act in one way but default to another, for example, failing to adopt the decision-maker role in urgent situations or neglecting to seek consultation when the IP team is working toward a shared understanding of the patient’s status. This reinforces the notion that identity positions – or leadership positions – are not fixed, secure roles suitable for every context [18]. Rather, they allow SPs to navigate the varying leadership expectations inherent in their work.

The identity positions should not be viewed as hierarchical or ranked; none is inherently better than the others since medical leadership takes different forms in different situations [35]. Different types of physician work and leadership are needed at different moments in IP care. Being a skillful physician and leader is more about adapting leadership to meet situational expectations than adopting a single, fixed leadership role as part of one’s professional identity [28,35]. Furthermore, none of the five positions was emphasized more than the others. In fact, multiple positions were often adopted within a single essay, in various combinations, illustrating how SPs shift between different positions throughout their day. This finding aligns with Williams et al. [28], who observed that residents move between positions during their training as they grow into the role of a physician. This demonstrates that SPs’ relationship with the IP team is not a fixed or predetermined role but rather a set of dynamic positions that are negotiated and shaped through team interaction.

### Two storylines

Two strong storylines emerged when examining the relations between different identity positions: IP teamwork as communication and IP teamwork as an organizational tool. These storylines have been identified in a medical context in previous studies as well [28,36]. Moreover, the heroic and collaborative narratives that were recognized by Berghout et al. [6] resonate with these storylines. This shows that the conflict between working interprofessionally and relying on teamwork and collaboration instead of the strong hierarchical structure of HC organizations is present in medical professions’ everyday work.

In the storyline of teamwork as communication, the entire team participates in negotiating what needs to be done regarding patient care and in shaping the nature of their collaboration. Resembling this storyline, Williams et al. [28] found that SPs’ learning experiences are shaped by their interactions with patients, nurses, and supervisors, thus highlighting the importance of teamwork and collaboration over organizational hierarchy. Not all professionals should perform the same tasks but everyone could have the opportunity to participate in and influence the collaborative process. When each team member has a voice, they also have the potential to shape the team dynamic. This corresponds to Møller et al. [36], who found that promoting a sense of equality is a dominant storyline in traditional HC morning reports.

By contrast, the storyline of teamwork as an organizational tool positions the SP as the most powerful holder of interactional rights and responsibilities, particularly within the decision-maker role. The SP assumes the authority to shape teamwork and care processes to their vision while also bearing responsibility not only for their own actions but for the outcomes of the entire team. This approach reinforces the hierarchical structure of HC organizations and mirrors findings from previous studies (e.g [28,36], that emphasize how hierarchical structures are sustained through interaction. For example, Møller et al. [36] have identified ‘preserving hierarchy’ and ‘making the chain of command visible’ as key themes in morning reports, aligning closely with the storyline of teamwork as an organizational concept.

### Team-centricity vs. physician-centricity

**Figure 1.**
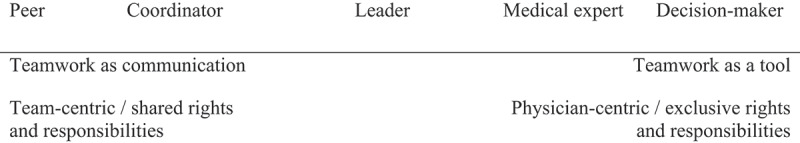
The continuum of the two distinct storylines and how responsibilities and rights are distributed across identity positions.

The five identity positions span a continuum from a fully team-centric position with shared rights and responsibilities to a strongly physician-centric position characterized by an authoritarian allocation of these rights and responsibilities. This tension between team and physician centricity was observed through both organizational and professional meanings. Organizational meanings are associated with administrative leadership roles within IP teams, while professional meanings relate to holding the highest level of medical expertise. Through this lens, we can map a progression from team-centric to physician-centric positions, along with a shift from shared to exclusive rights and responsibilities. The relationship between these two storylines, the five positions, and the continuum from physician-centricity to team-centricity is presented in Figure 1.

When SPs adopt team-centric identity positions, both their focus and that of the team shift towards providing optimal patient care. Conversely, within physician-centric identity positions, SPs’ responsibilities extend beyond patient care to include team management and process oversight. This dynamic illustrates the tension between organizational hierarchy and collaboration among IP team members, as highlighted by the study’s storylines and previous research (e.g [6,28,36]. Physician-centric positions do not dismiss patient-centeredness; rather, SPs emphasize patient responsibility in all identity positions.

Furthermore, organizational structures can support both team-centric and physician-centric positions. Strict protocols for non-negotiable situations (e.g., resuscitation) reinforce physician-centricity at the organizational level, while fostering interprofessional communication supports team-centricity. However, these positions are not entirely distinct; increased awareness of their interplay is advocated. Ultimately, a physician’s leadership role involves balancing and shifting between physician-centered and collaborative positions, depending on the leadership expectations and needs in various IP care situations.

### The dual-identity question

The diversity of identity positions highlights how flexibly and dynamically SPs construct their professional identity and find their own way of embodying the leadership expected of them during specialization training [4,37]. Thus, our results support the claims made by Ahuja et al. [38] and Kyratsis et al. [39] regarding the fluid, evolving nature of professional identity. Previous studies have suggested that physicians struggle with a dual identity, namely, integrating leadership into their professional identity (e.g [6–9,17]. However, the findings of this study suggest that the question is not *whether* SPs accept or reject leadership as a part of their professional identity but rather *how* they incorporate it. Our findings propose that, in IP teams, SPs explore various ways of engaging with leadership through navigating and adopting different identity positions. We argue that framing the issue as a matter of ‘dual identity’ oversimplifies the question and the broader issue of professional identity. Instead, leadership should be viewed as a flexible component of an SP’s professional identity.

By becoming aware of their positions, physicians can actively influence how these roles are negotiated, choosing whether to relate to IP teams primarily as a leader, a representative of the organization, or a peer within the team. Understanding physician positioning opens up new perspectives on leadership identity as a dynamic, relational, and communicative process rather than a fixed role. This perspective also allows for a broader understanding of physician identity, one that includes a fluid and adaptable leadership component. Even when appointed as team leaders, physicians can draw on context-appropriate identity positions and help develop IP communication toward more inclusive and collaborative practices.

### Physician education

Previous studies have consistently shown that SPs feel their education does not sufficiently prepare them for the leadership responsibilities expected in their profession (e.g [4,17]. Despite the inclusion of management and communication topics in the medical curriculum, some SPs reported feeling underprepared for real-world leadership demands, suggesting a potential gap between training content and clinical practice. Our findings suggest that medical education should recognize SPs’ professional identity formation as a multidimensional and dynamic process, in which meanings of medical leadership are also constructed. Therefore, it is of vital importance to reflect on how medical leadership is introduced and reinforced during the whole trajectory of medical education. We argue that adopting a socio-constructionist approach to physician’s identity and to PIF would help SPs to cope with the pressures that emerged from the leadership role. From a socio-constructionist perspective, identities are constructed through discourse and positioning, shifting the focus from individual traits to interaction [7]. Furthermore, we suggest that the importance of communication in collegial relations, management, and teamwork would be recognized beside the importance of patient-physician relationship.

Furthermore, the development of medical leadership identity should be integrated into SPs’ professional identity from the outset of their training. This means that topics such as IP communication, teamwork, and leadership should be incorporated throughout all stages of medical education, from undergraduate studies to residency and specialization training. Leadership identity cannot simply be added to professional identity at the end of training. Since PIF is enacted through interactions and IP team communication, strong IP team communication skills must be emphasized throughout medical education.

## Strengths and limitations

This study offers a portrayal of a specific moment in the SPs’ professional identity formation process, illustrating how SPs construct their leadership identity as an integral part of their professional identity. The analysis was conducted and reported in detail, and the findings are considered robust, as the identified positions and storylines can be traced back to multiple speech acts. The overall reliability of the data is strong, given that it includes participants from a wide range of specialties, from psychiatry to surgery, and represents students from hospitals and primary health care. Participant recruitment for this exploratory study was inclusive, with all students enrolled in the communication module given the opportunity to participate. The variety of the positions and the resemblance with earlier studies suggest that these findings are transferrable at least to other SPs in Finnish medical education. Additionally, the study benefits from the expertise of the researchers, who have substantial experience in leadership programs in medical education and qualitative research methodologies, lending further credibility to the analysis and interpretation of the data.

A limitation lies in the reliance on reflective text rather than naturally occurring interaction data and observation, even though PT and positioning analysis were applied [18]. The study is based on the analysis of essays, thus providing a ‘snapshot in time’ of SPs’ views and conceptions. While the dataset may not be considered traditional, it is not uncommon in positioning analysis (see e.g., [26, 40], and it offers a particularly clear view of how early career physicians perceive their roles within IP teams. Nevertheless, it is important to note that the analysis is based solely on physicians’ perspectives. As such, it may not accurately reflect actual interactions. This study serves as a prime exploration of what a positioning analysis dataset might entail, and future research could usefully explore how IP team members view the role of a physician.

A notable limitation of this study is that we were unable to analyze the data based on participant individual factors, which may have influenced the interpretation of leadership positions and interactions. The dataset consists of responses from only one semester of students. Including students from multiple semesters would strengthen the credibility of the data and transferability of the findings. Another limitation is the lack of systematically collected background information about participants. It would have enabled more versatile implications and a more comparative approach. Nonetheless, such a comparative analysis was beyond the scope of this study and did not compromise the current analysis.

Additionally, we were not able to fully evaluate the participant selection process or determine whether the inclusion or exclusion of certain participants may have influenced the findings. Although all students enrolled in the communication module were invited to participate, it remains possible that self-selection or non-participation introduced bias. We acknowledge this as a limitation. In future studies more controlled or stratified sampling approaches would enhance representativeness and reduce potential bias.

## Conclusion and recommendations

This study reveals that physician leadership within IP teams is a dynamic, context-sensitive process shaped by fluid identity positions – peer, coordinator, team leader, medical expert, and decision-maker – each reflecting varying degrees of team- or physician-centricity. These positions span a continuum from shared to exclusive rights and responsibilities, influenced by both organizational structures and professional expertise. Rather than viewing leadership as a fixed or hierarchical role, the findings emphasize its relational and communicative nature, negotiated through daily team interactions. The study challenges the notion of a dual-identity struggle by showing that SPs do not simply accept or reject leadership but actively construct it as a flexible component of their professional identity. Two overarching storylines – teamwork as communication and teamwork as an organizational tool – highlight the tension between collaboration and hierarchy in clinical practice. These insights underscore the need for medical education to embed leadership, teamwork, and communication training throughout all stages of professional development, supporting identity formation as a socio-constructivist, interaction-driven process.

We recommend that future research focus on exploring the tensions between various situational leadership expectations in physicians’ work and how these tensions influence their PIF. While there have been commendable efforts to examine how physicians perceive teamwork [41], further exploration is warranted. Specifically, future studies should ethically investigate naturally occurring interactions and communication within IP teams. This can be achieved through methods such as non-intrusive ethnographic observation, audio or video recordings with informed consent, and anonymized discourse analysis, all conducted under strict ethical guidelines. Additionally, we suggest examining how communication perspectives are shaped and evolve during medical school and specialization training. For routine practice, we recommend implementing structured reflection sessions, interprofessional simulation training, and mentorship programs to support physicians in navigating leadership tensions and fostering collaborative communication skills.

## Aknowledgements

We want to sincerely thank Specialized Doctor of General Medicine, Emmi Lautamatti for her insightful comments and contribution to this manuscript.
